# Polymer Modification of Surface Electronic Properties of Electrocatalysts

**DOI:** 10.1021/acsenergylett.2c00199

**Published:** 2022-04-04

**Authors:** Anirudh Venugopal, Laurentius H. T. Egberts, Jittima Meeprasert, Evgeny A. Pidko, Bernard Dam, Thomas Burdyny, Vivek Sinha, Wilson A. Smith

**Affiliations:** † Materials for Energy Conversion and Storage (MECS), Department of Chemical Engineering, Faculty of Applied Sciences, Delft University of Technology, Delft 2629HZ, The Netherlands; ‡ Inorganic Systems Engineering (ISE), Department of Chemical Engineering, Faculty of Applied Sciences, Delft University of Technology, Delft 2629HZ, The Netherlands; § Renewable and Sustainable Energy Institute (RASEI), University of Colorado Boulder, Boulder, Colorado 80303, United States

## Abstract

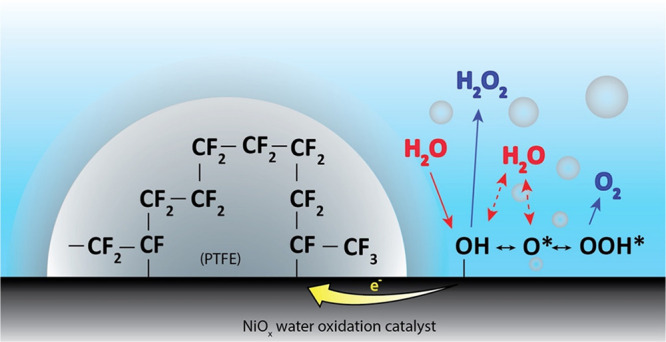

Finding alternative ways to tailor the electronic properties of a catalyst to actively and selectively drive reactions of interest has been a growing research topic in the field of electrochemistry. In this Letter, we investigate the tuning of the surface electronic properties of electrocatalysts via polymer modification. We show that when a nickel oxide water oxidation catalyst is coated with polytetrafluoroethylene, stable Ni–CF_*x*_ bonds are introduced at the nickel oxide/polymer interface, resulting in shifting of the reaction selectivity away from the oxygen evolution reaction and toward hydrogen peroxide formation. It is shown that the electron-withdrawing character of the surface fluorocarbon molecule leaves a slight positive charge on the water oxidation intermediates at the adjacent active nickel sites, making their bonds weaker. The concept of modifying the surface electronic properties of an electrocatalyst via stable polymer modification offers an additional route to tune multipathway reactions in polymer/electrocatalyst environments, like with ionomer-modified catalysts or with membrane electrode assemblies.

The electrochemical conversion of abundant feedstocks such as water, CO_2_, N_2_, and O_2_ to green value-added chemicals using renewable electricity is very attractive from a sustainability perspective as these chemicals can serve as the basic feedstock to the chemical industry, replacing fossil-based resources. As a result, there has been significant research interest into electrochemical conversion technologies over the past decades.^1−4^

Across the various electrochemical technologies, identifying the right electrocatalyst for different reactions of interest has been one of the main focus areas of researchers. The Sabatier principle is often used as a guideline to find a suitable catalyst for a particular reaction.^5,6^ Based on this principle, computational, and more recently, machine learning approaches have been used to predict potential candidates for selectively catalyzing different reactions.^6−8^ When a singular material’s properties is insufficient for good catalytic activity, the properties of a catalyst or substrate can be tuned by mixing/alloying different elements within the periodic table.^9,10^ However, such strategies may also affect the bulk material properties of the catalyst, such as the conductivity, which is undesirable. Additionally, degradation or phase separation of mixed/alloyed catalysts and *operando* catalyst restructuring often occur under reaction conditions, making them ineffective for long-term operation in practical applications.^11−13^ Therefore, there is a need for alternative strategies to tailor the surface electronic property of a catalyst to make it more selective and efficient toward the reaction of interest, while also not affecting their bulk properties.

Polymer modification was previously shown to alter the surface electronic properties of metallic sensing materials in optical hydrogen sensors.^14,15^ This concept could be extended to the field of electrochemistry. Polymers have been previously used in the field of electrochemistry to modify the electrode/electrolyte interfaces;^16,17^ however, their effect on the surface electronic properties of the electrocatalysts was not extensively studied before. In this Letter, we discuss the modification of surface electronic properties of electrocatalysts upon polymer loading, using the recent example of polymer-coated water oxidation catalysts.^16^ Recently, Xia *et al.*(16) demonstrated that the selectivity of common water oxidation reaction catalysts can be altered from the four-electron water oxidation reaction to oxygen to the two-electron water oxidation reaction to hydrogen peroxide by coating these catalysts with a hydrophobic polytetrafluoroethylene (PTFE) polymer. The NiO_*x*_/PTFE system is shown in Figure 1. It was suggested that this change in selectivity was due to the weakening of the binding energy of the OH* intermediate by two factors: (1) the destabilization of the OH* intermediate due to the breakage of the hydrogen bonding network within the surrounding electrolyte because of the presence of the hydrophobic PTFE and (2) a less oxidized catalyst surface due to the reduced local H_2_O concentration in the presence of hydrophobic PTFE. Theoretical calculations showed that this less oxidized catalyst surface can weaken the OH* binding energy, altering the selectivity of the water oxidation reaction. While these are plausible explanations for the effect of PTFE, more work needs to be done to experimentally verify and support these claims, as well as providing further details that allow the approach to be extended to other electrochemical applications.

**Figure 1:**
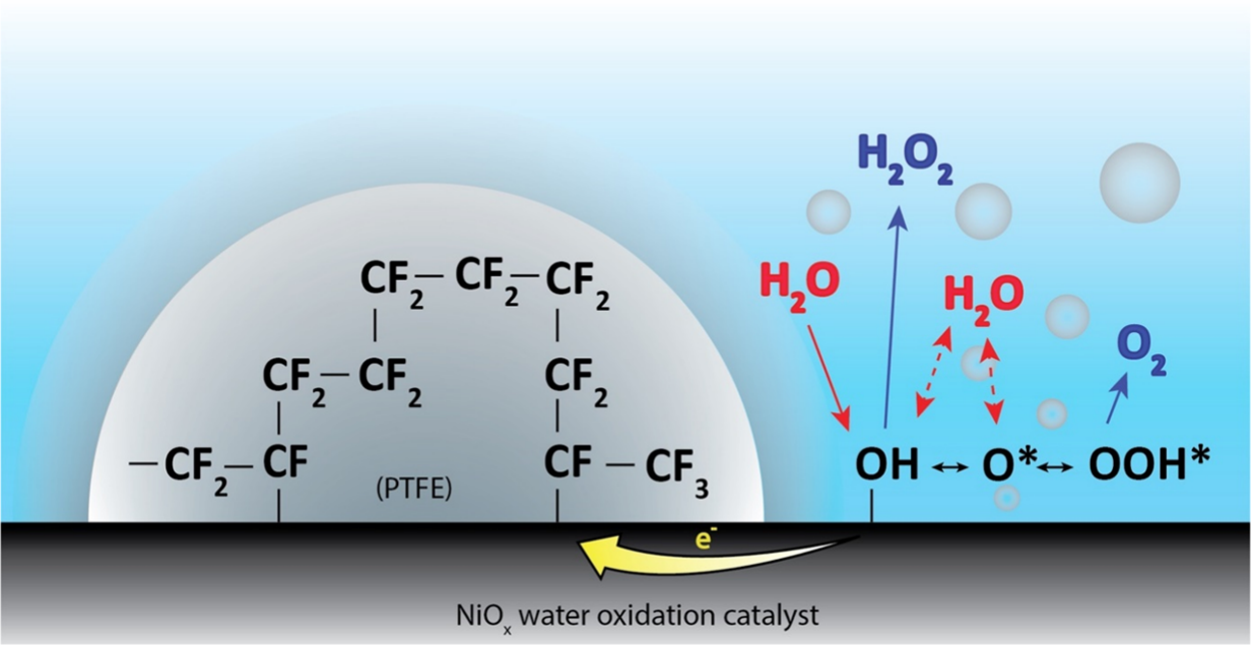
Schematic of the PTFE polymer-coated NiO_*x*_ water oxidation catalyst system. The two-electron and the four-electron water oxidation pathways to hydrogen peroxide (H_2_O_2_) and oxygen (O_2_) are also shown here. Red represents the reactants, and blue represents the products. The PTFE polymer is chemically bound to the NiO_*x*_ water oxidation catalyst. The electron-withdrawing nature of the chemically bound fluorocarbon molecules alters the catalytic properties of adjacent active sites.

In this work, we present an alternative explanation for the effect of this PTFE loading for the stable modification of an electrocatalyst’s surface properties. We show that polymers having an electron-withdrawing or donating character can alter the electronic properties of the adjacent active sites in the catalyst when bound to the catalyst surface. Using X-ray photoelectron spectroscopy (XPS), we show that the PTFE-coated nickel foam results in the formation of Ni–CF_*x*_ bonds at the nickel oxide/PTFE interface. Further, using density functional theory (DFT) calculations on a CF_*x*_ bound β-NiOOH surface, we demonstrate that the binding of OH* intermediate is destabilized in the presence of these Ni–CF_*x*_ bonds, while the step in Gibbs free energy toward the O* intermediate is increased. The reduced binding energy of OH* and the suppressed formation of O* intermediate collectively tunes the selectivity of the water oxidation reaction toward the two-electron pathway of hydrogen peroxide formation.

We start by experimentally reproducing the work of Xia *et al.*(16) on Ni foam, by coating the Ni foam with PTFE using the same procedure as their work and studying the changes in selectivity toward the two-electron water oxidation reaction. These results and their explanations are presented in the Supporting Information (Figures S1 and S2). In short, an increased selectivity toward the two-electron water oxidation reaction to hydrogen peroxide was reproduced when the Ni foam was coated with PTFE, similar to the observations of Xia *et al.*,^16^ confirming that the polymer modification has indeed altered the catalytic property of the electrocatalyst.

To elucidate the cause of the observed selectivity change upon polymer loading, X-ray photoelectron spectroscopy (XPS) was performed on pristine and PTFE-coated Ni foam electrodes. A Mg Kα X-ray source was used to perform XPS measurements, to prevent the overlap of the Ni 2p 3/2 core electron spectra and the fluorine KLL auger electron spectra, as shown in Figure S3. Through the comparison of the Ni 2p 3/2 spectra in Figure 2a, the pristine and PTFE-coated Ni electrodes show large differences in their relative peak positions and shapes, suggesting that the PTFE coating has introduced electronic modifications on the surface nickel atoms. Under normal conditions in air, Ni foam is covered with a native oxide layer, in the form of NiO.^20^ This native oxide layer is typically ∼2–3 nm thick, which is also the probing depth of the XPS. The spectrum of pristine Ni foam in Figure 2a is thus a typical Ni 2p 3/2 spectrum for a NiO layer, with the main Ni 2p 3/2 peak at 853.9 eV along with its broad satellite peaks at 860.9 eV. Additionally, the Ni 2p 3/2 main peak also has a shoulder peak at 855.5 eV. The most common interpretation suggests that this shoulder peak is a result of some surface and nonlocal screening effect.^21,22^ The spectrum for the PTFE-coated Ni foam sample in Figure 2a is very different compared to that of the pristine Ni sample. The Ni 2p 3/2 main peak in the PTFE-coated sample has shifted to higher binding energies while its satellite peak is relatively unchanged when compared to that of the pristine sample. Additionally, the Ni 2p 3/2 main peak now has two shoulder peaks at 853.3 and 853.9 eV. The broad shoulder peak present in the pristine sample at 855.5 eV now overlaps with the main peak of the PTFE-coated sample. For both spectra, a small shoulder peak at 852.4 eV is also visible, which is the contribution from the bulk nickel metal.

**Figure 2:**
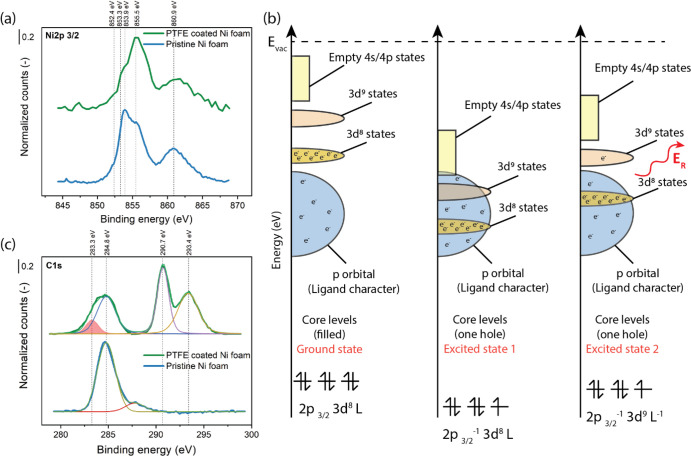
(a) Ni 2p 3/2 XPS spectra of the pristine and PTFE-coated Ni foam water oxidation catalyst. (b) Schematic representation of Kotani–Toyozawa model^18,19^ in nickel insulators. Here, the ground state and the two different excited states are shown. In the ground state, the metal d band is above the ligand p band. In the excited state, because of the photoionized ion, the d band is pulled below the Fermi level. Now, there is a possibility of the 3d^9^ band getting an electron from the ligand p band, resulting in local screening. When this d band is not filled, it results in the excited state 1, which is represented by the satellite peak in the Ni 2p 3/2 spectra. When the d band is filled by an electron from the ligand p band, it results in the excited state 2, which is represented by the main peak in the Ni 2p 3/2 spectra. (c) C 1s spectra of the pristine and PTFE-coated Ni foam sample. The fitted peak with the red fill represents the Ni–C bond in the C 1s spectra.

To explain the changes in the main peak position in the Ni 2p 3/2 spectra with and without the PTFE coating, we use the Kotani and Toyozawa model.^18,19^ The ejection of a core electron creates an instantaneous increase in the Coulombic potential around the photogenerated core hole. This localized increase in the potential pulls the metal orbitals toward the nucleus of this photoionized ion, as shown in Figure 2b. As a result, the empty 3d^9^ band in nickel is locally lowered below the top of the valence band, shown as excited state 1 in Figure 2b. This excited state is a transition state that exists only within the lifetime of the core hole, which is in the order of femtoseconds. During this short period, there is a finite probability of this 3d^9^ band being filled by the electrons from the top of the valence band. In cases where the 3d^9^ band remains empty, the energy of the emitted photoelectron is not altered and results in the higher binding energy satellite peak in the Ni 2p 3/2 spectra. In cases where the 3d^9^ band is filled (shown as excited state 2 in Figure 2b), energy is released because of a relaxation process and is then transferred to the emitted photoelectron. This process increases the kinetic energy of the photoelectron, resulting in the main peak at a lower binding energy in the Ni 2p 3/2 spectra. The valence band in these insulating materials primarily has a ligand characteristic. The magnitude of relaxation energy is thus dependent on the nature of the ligand p band. Therefore, the binding energy of the Ni 2p 3/2 main peak in insulating Ni materials like NiO is affected by the nature of the ligand coordinated to the Ni, while the binding energy of the Ni 2p 3/2 satellite peak is not affected by the nature of the ligand. The difference in the main peak positions in the Ni 2p 3/2 spectra in the pristine and the PTFE-coated Ni samples is thus an indication that a different Ni-ligand coordination exists in the two samples. The Ni 2p 3/2 satellite peak positions of the pristine and PTFE-coated samples remain unchanged because these peak positions are not affected by the nature of the ligand, validating this ligand theory. The valence band spectra of the pristine and PTFE-coated samples in Figure S4a also confirm that the nature of the valence band has been altered with the PTFE coating, showing additional contributions from the PTFE layer.

These results indicate that the PTFE polymer is electronically coordinated to the Ni in the PTFE-coated samples. This coordination can either be through the carbon or through the fluorine atoms in the PTFE polymer. If the coordination was through the fluorine atoms, there should also be signatures of the Ni–F bond in the Ni 2p 3/2 spectra and in the F 1s spectra (Figure S4b). The main peak for Ni–F bond is expected to be around ∼858 eV in the Ni 2p 3/2 spectra,^22,23^ which is not present in Figure 2a. No Ni–F bond feature was found in the F 1s spectra either. On the other hand, if a Ni–C bond is present, a peak is expected in the region of ∼853.3 eV in the Ni 2p 3/2 spectra.^24−26^ This is one of the shoulder peaks present in the Ni 2p 3/2 spectra for the PTFE-coated sample, suggesting that the polymer is linking with the nickel center through the carbon, resulting in Ni–CF_*x*_ bonds. The shoulder peak at 853.9 eV in the Ni 2p 3/2 spectra originates from the Ni–O coordination from the bulk, which is not affected by the CF_*x*_ ligand coordination at the surface of the PTFE-coated samples. The Ni–C coordination is further confirmed by the C 1s spectra, shown in Figure 2c, where an additional shoulder peak at 283.3 eV is observed upon PTFE coating, which is typically ascribed to a Ni–C bond in the C 1s spectra.^24,27^ Additionally, peaks pertaining to CF_2_ and CF_3_ originating from the PTFE polymer are visible at 290.7 and 293.4 eV in the C 1s spectra of the PTFE-coated sample.^15,28^ Adventitious carbon (C–C) and (O–C=O) peaks at 284.8 and 287.8 eV are also visible for the pristine and PTFE-coated samples, respectively. The F 1s XPS spectra of the sample after electrolysis, in Figure S4b, also confirm that the polymer did not change or degrade during the electrolysis.

Having confirmed the existence of the Ni–polymer bond on the PTFE-coated sample via XPS, we proceed to further understand its impact on the selectivity toward the water oxidation reaction using computational techniques. The activity and selectivity changes on different catalysts can be predicted by studying the changes in the free energy of the reaction intermediates. The adsorption free energies of the relevant water oxidation intermediates (Δ*G*_OH*_, Δ*G*_O*_, and Δ*G*_OOH*_) can be calculated using the density functional theory (DFT) calculations.^29−31^ Although the thermodynamic analysis can only be taken as qualitative, because kinetic activation barriers between the intermediates are not included, it has proven useful in rationalizing trends in activity for catalytic surfaces.^32,33^ Therefore, performing DFT calculations on the pristine and PTFE-coated Ni foam electrodes can help us understand the effect of the Ni–polymer bond on the water oxidation reaction selectivity. The presented thermodynamic analysis is thus a first step toward understanding the activity and selectivity changes on polymer modified electrocatalysts.

Considering the recent investigations from Carter *et al.*,^29,30,34^ we chose the β-NiOOH structure of the catalyst with a staggered arrangement of intercalated protons for the computational investigations. The details of the computational modeling methods are described in the Supporting Information. The Perdew–Burke–Ernzerhof (PBE) functional with projector augmented wave (PAW) potentials was used. The DFT+U correction method of Dudarev *et al.*(35,36) was employed to improve the known deficiencies of generalized gradient approximations (GGA) functionals when describing partially occupied 3d shells. A U–J value of 5.5 eV for Ni(III) was added in combination with the PBE exchange–correlation functional. This value was adapted from the linear response theory calculations of Li and Selloni on β-NiOOH and has been confirmed to lead to accurate replication of electronic and structural properties among other parameter values by Carter *et al.*(29,37,38) To model the PTFE-coated catalyst surface, we introduce a Ni–polymer bond in the model via CF_3_ fragments which were coordinated to the nickel by removing a terminating OH group from a Ni site in each unit cell (Figure S5c). Some of the possible intermediates of the water oxidation reaction (OH*, O*, and OOH*)^29,39^ were introduced at the coordinatively unsaturated Ni sites for both the β-NiOOH and β-NiOOH–CF_3_ unit cells, as shown in Figure 3a–d. We investigated both atop and bridging binding modes for all intermediates. The bridging mode binding was found to be most stable for all intermediates. The free energy of steps involving the formation of H^+^ and e^–^ were obtained by referencing it to the free energy of H_2_ using the standard hydrogen electrode (SHE, pH, *p* = 1 atm, *T* = 298 K).^40^

**Figure 3:**
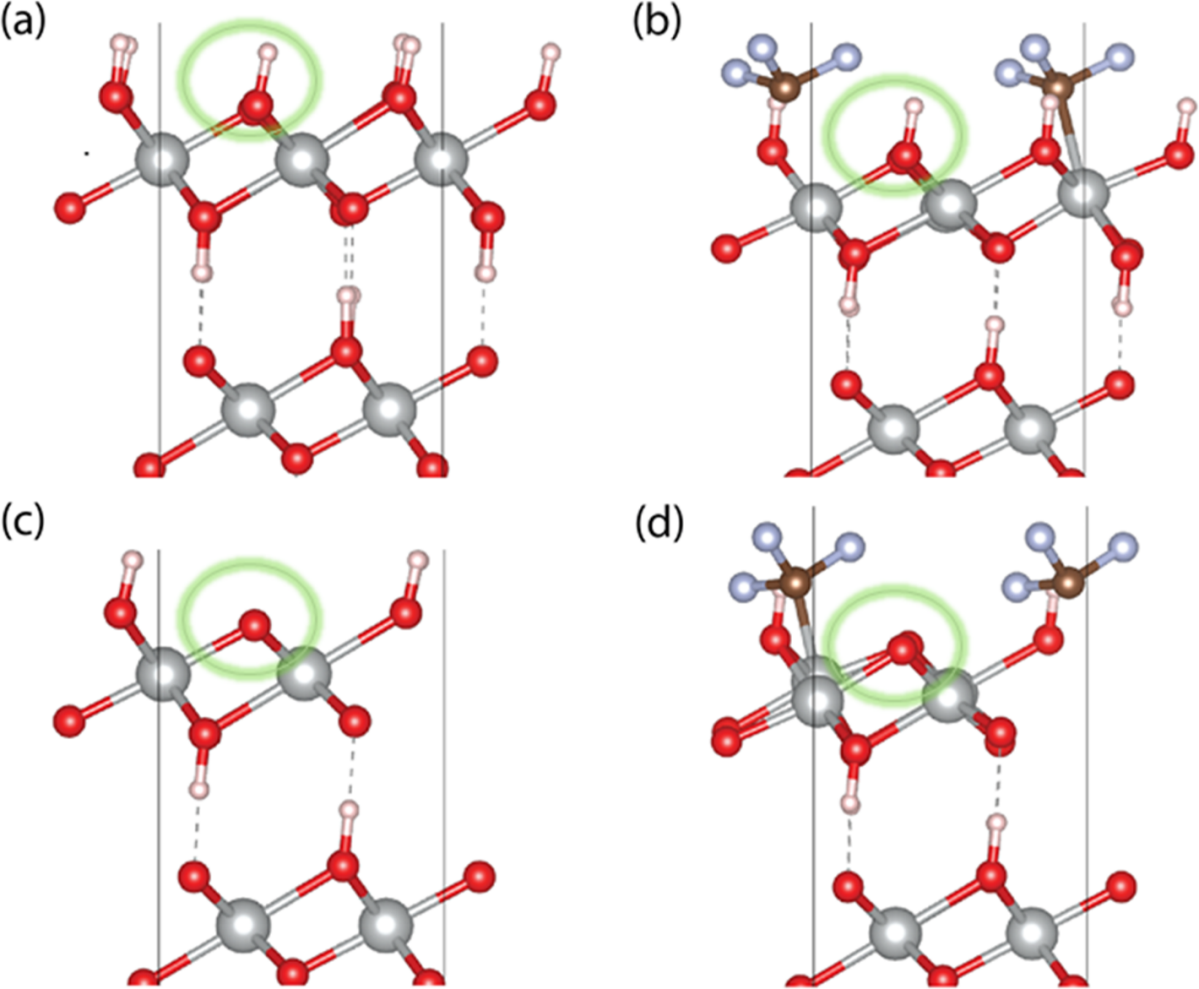
β-NiOOH and β-NiOOH–CF_3_ unit cells with OH* (a and b) and O* (c and d) water oxidation reaction intermediates. The adsorbed intermediates are highlighted in green circles to guide the readers. Color code for atoms: O, red; Ni, gray (larger spheres); F, blue; C, brown; H, white. The Ni–C distances for OH* and O* β-NiOOH–CF_3_ slabs are 2.34 and 2.06 Å, respectively.

We considered stepwise mechanisms releasing (H^+^ + e^–^) pairs for both the 4e^–^ and 2e^–^ pathways. For the 4e^–^ pathway the water oxidation reaction proceeded via OH*, O*, and OOH* intermediates forming O_2_(g) and 4(H^+^ + e^–^) as the products (eqs 1, 2, 5, and 6 excluding eqs 3 and 4).^31,39,41−43^
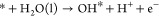
1
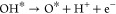
2
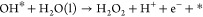
3
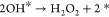
4
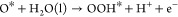
5
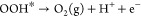
6

The mechanism for the 2e^–^ pathway consists of eq 1 and eq 3 or 4, which results in H_2_O_2_ as product alongside two pairs of (H^+^ + e^–^). The first step in both mechanisms is the formation of OH* releasing a (H^+^ + e^–^) pair (Figure 4a). In the 4e^–^ pathways, a subsequent electrochemical step results in O* species with a second (H^+^ + e^–^) pair (eq 2). Next, the nucleophilic addition of water to O* results in O–O coupling along with the release of a third (H^+^ + e^–^) pair forming the OOH* species (eq 5). The OOH* species forms O_2_(g) product and releases the fourth and final (H^+^ + e^–^) pair (eq 6). In the 2e^–^ pathway, the OH* species does not convert to O* but instead undergoes O–O coupling either by a nucleophilic addition to water via eq 3 (Volmer–Heyrovski) or via coupling of two OH* species (eq 4, Volmer–Tafel) (also see Figure 4b).

**Figure 4:**
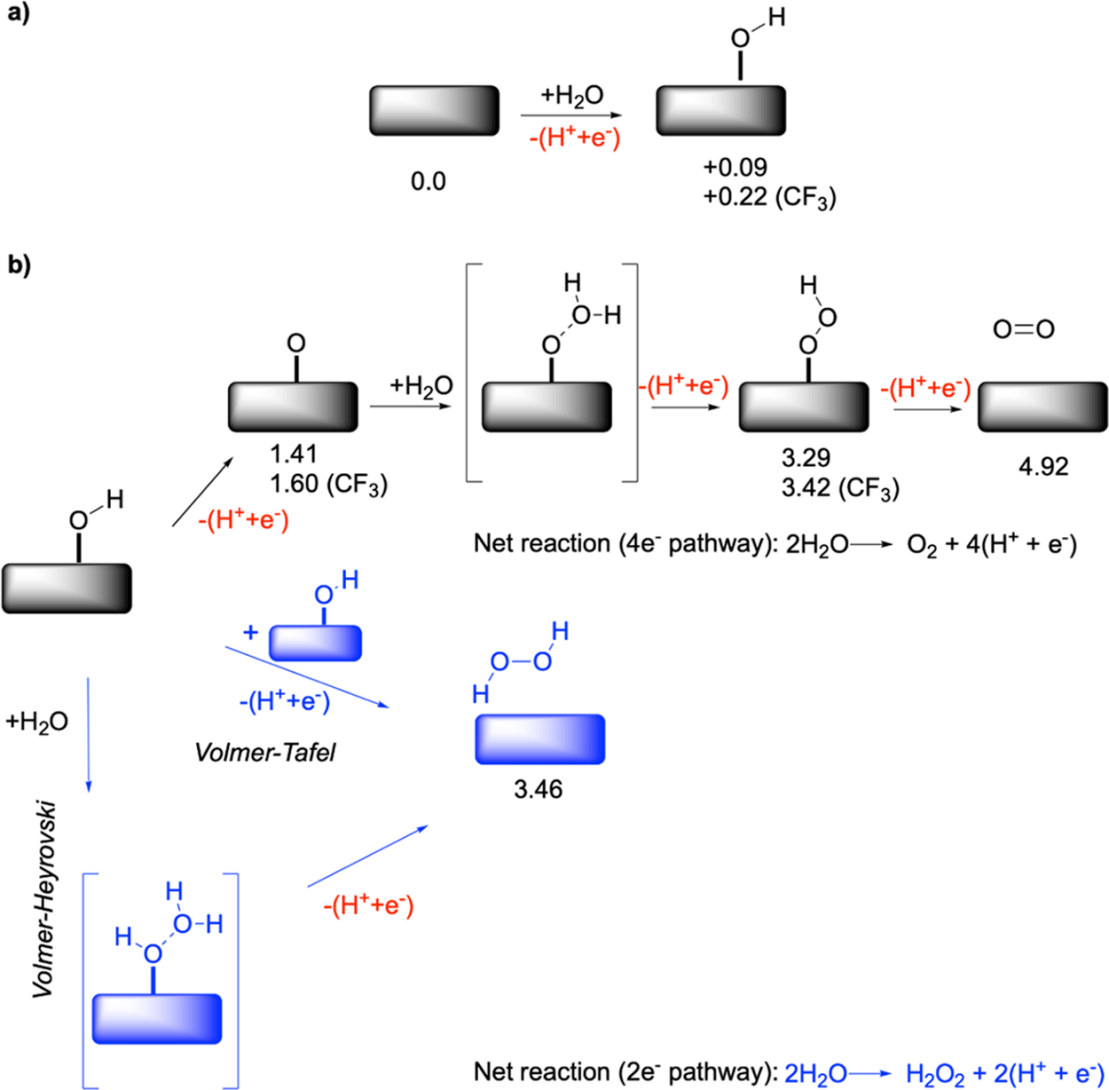
(a) Formation of OH* species, which is a descriptor of selectivity for the 4e^–^ versus the 2e^–^ pathway for the water oxidation reaction. The DFT-computed Gibbs free energy (eV) is shown below for the pristine and the CF_3_-coated slabs. All the calculations were performed at *U* = 0 V. (b) Further steps in the mechanism leading to O–O coupling and eventual production of O_2_ or H_2_O_2_ via the 4e^–^ (black) and 2e^–^ (blue) pathways. For the 2e^–^ pathway for the Volmer–Tafel and Volme–-Heyrovski mechanisms are shown. The water adducts shown in brackets highlight the nucleophilic addition of water to O* (4e^–^) and OH* (2e^–^) adducts and do not necessarily represent the underlying transition states. The computed Gibbs free energy values of the other water oxidation intermediates for the pristine and CF_3_-coated samples are also shown below the respective slabs.

The results from DFT calculations are presented in the Supporting Information. Tables S1 and S2 contain the electronic energy, the zero-point energy, thermal corrections, the Gibbs free energies of intermediates, and the Δ*G* values for elementary steps in the mechanism, The cumulative Gibbs free energy is then plotted and shown in Figure S6. Compared to the bare surface slab, the binding of OH is destabilized by 0.13 eV for the β-NiOOH–CF_3_ surface. A weakening of OH* Gibbs free energy indicates a shift from the four-electron pathway toward the two-electron route.^31,44,45^ A Bader charge analysis was also performed on the above systems to investigate the electronic effect of CF_3_. The results from Bader charge analysis are presented in Table S3. The sum of Bader net atomic charges on the OH* complex is zero, as expected for a charge neutral unit cell. For the β-NiOOH–CF_3_ surface, the sum of Bader net atomic charges on all atoms except the CF_3_ unit is +0.30, indicating the strong electron-withdrawing effect of CF_3_. Further analysis of Bader net atomic charges shows that the OH moiety in OH* is more electropositive by 0.09 units on the β-NiOOH–CF_3_ surface. This should further facilitate nucleophilic addition of a water molecule forming the H_2_O_2_ product via an O–O coupling step (Figure 4b) and mitigate immediate further oxidation toward the O* intermediate as in the 4e^–^ pathway. This is reflected in  eV for the β-NiOOH–CF_3_ surface, which is 0.06 eV higher than that for the β-NiOOH surface (see Figure S6). Therefore, the presence of CF_3_ units modifies the surface electronic property by a strong electron-withdrawing effect and lowers the propensity toward the 4e^–^ pathway.

We note that a OH* binding energy of 0.22 eV, which should ideally be 1.77 eV,^31^ is still rather low for a highly active and selective material for the 2e^–^ pathway. We have used a rather simple model to mimic the PTFE coating by a chemically bound Ni–CF_3_ per unit cell. This simplified model already provides qualitative insights into the promoting role of PTFE units toward the selectivity for 2e^–^ pathway. The presence of PTFE coating can also influence the interaction of surface adsorbed intermediates such as OH* with water, which can in turn influence the kinetics of H_2_O_2_ formation via the Volmer–Heyrovski mechanism. Ni sites that are next to PTFE layer and sites that are farther away can have different binding affinities to OH* and can influence the H_2_O_2_ production via the Volmer–Tafel mechanism. Incorporation of such effects would require a more rigorous computational treatment possibly via (*ab initio*) molecular dynamics simulations, which is beyond the scope of the present work. The present model nonetheless captures the molecular effect of the PTFE coating toward promoting the 2e^–^ pathway via an electron-withdrawing effect which destabilizes the OH* intermediate. This weakening of the binding energy of the OH* intermediate directly explains the experimentally observed change in selectivity toward the two-electron hydrogen peroxide reaction on PTFE-coated samples.

In principle, the strategy of modification of the surface electronic character of the electrocatalyst and subsequent tuning of the reaction selectivity, upon polymer loading, can be extended to other electrocatalytic reactions and systems. Even though polymers have previously been used to modify electrocatalysts, by influencing the reaction environment,^46,47^ their effect on the electronic properties of the catalyst has not been extensively investigated before. There are several articles in the literature that demonstrate different instances where a polymer is bound to an electrocatalyst surface.^48,49^ However, any change in the electrocatalyst performance because of this polymer coating was normally attributed to the catalyst site poisoning due to this polymer binding. Through this work, we show that this polymer binding can induce additional surface electronic changes on the electrocatalyst. A thorough understanding of this concept becomes important with the increased usage of polymer/electrocatalyst interfaces, in the form of ionomer-coated catalysts and solid-state electrolytes.^17^ This is especially important with multipathway reactions, like CO_2_ reduction, where a small change in the surface electronic property can alter the selectivity of different reaction pathways. In the current context, with a careful selection of the polymer, the Ni foam can be made more favorable or less favorable for the four-electron water oxidation reaction compared to the two-electron formation of hydrogen peroxide.

In this work, we study the modification of the surface electronic property of nickel-based water oxidation catalysts upon polymer loading and use it to explain the change in the water oxidation reaction selectivity on PTFE-coated Ni foam catalysts. Using XPS, we show that upon coating these catalysts with the PTFE polymer, stable Ni–CF_*x*_ bonds are formed at the nickel oxide/PTFE interface. Further using DFT calculations on β-NiOOH and β-NiOOH–CF_3_ structures we show that, because of the electronegativity of the fluorine atoms, the CF_3_ group withdraws the electrons from the oxygen atom of the adsorbed OH* intermediate. This electron-withdrawing effect of the CF_3_ group weakens the binding energy of the OH* intermediate and makes it more difficult to form the adsorbed O* intermediate. The weakening of the OH* intermediate makes it easier to take the two-electron pathway to H_2_O_2_, while the increased energy requirement to form the O* intermediate suppresses the four-electron pathway to oxygen. Therefore, this dual effect of favorable H_2_O_2_ formation and suppressed OER pathway on PTFE-modified water oxidation catalysts explains the experimentally observed selectivity difference. In principle, this approach of tuning the electronic property of electrocatalysts with polymers with electron-withdrawing/donating character can be extended to other heterogeneous electrochemical systems.

## Supporting Information Available

Materials and methods, details of the DFT modeling, and additional experimental results and discussion (PDF)
