# Supplementary material for: Polymer Modification of Surface Electronic Properties of Electrocatalysts
Source: ACS Energy Lett. 2022 Apr 4;7(5):1586–93. doi: 10.1021/acsenergylett.2c00199 (PMC9112331; doi:10.1021/acsenergylett.2c00199)
Supplement: Supplementary file 1 — nz2c00199_si_001.pdf [file nz2c00199_si_001.pdf]

Polymer modification of surface electronic properties of electrocatalysts

Anirudh Venugopal[1], Laurentius H. T. Egberts[1], Jittima Meeprasert[2], Evgeny Pidko[2], Bernard Dam[1], Tom Burdyny[1], Vivek Sinha* [2], Wilson Smith* [1,3]

1. *Materials for Energy Conversion and Storage (MECS), Department of Chemical Engineering, Faculty of Applied Sciences, Delft University of Technology, Delft, 2629HZ, The Netherlands*
2. *Inorganic Systems Engineering (ISE), Department of Chemical Engineering, Faculty of Applied Sciences, Delft University of Technology, Delft, 2629HZ, The Netherlands*
3. *Renewable and Sustainable Energy Institute (RASEI), University of Colorado Boulder, Boulder, Colorado 80303, United States*

Supplementary information

Materials and methods

Preparation of PTFE dip-coated Ni foam

PTFE dip-coated nickel foams were fabricated by first ultrasonically cleaning (Emmi-30 HC sonicator) the 2.5 x 2.5 cm^2 substrate (0.5 mm Ni foam 110PPI, Gelon Energy Co. Ltd) consecutively in acetone and de-ionized water, facilitating or enhancing the removal of contaminants adhering to the three-dimensional metal scaffold. Substrates were dried with nitrogen gas. Then, the foam was pre-treated by UV/ozone cleaning (60 °C, 45 min). Ni foam was soaked horizontally into 60 wt% PTFE aqueous solution for 10 min and dried vertically at 120 °C under ambient conditions for 1 hour. Samples with lower concentrations of PTFE were prepared by diluting the 60% PTFE solution. The Ni foam-PTFE60 electrode was annealed in a tube oven at 350 °C under argon atmosphere for 30 min, unless otherwise specified.

Pristine Ni samples for blank measurements were also cleaned using the exact same procedure and annealed in a tube oven at 350 °C under argon atmosphere for 30 min, after dipping in ultrapure water for 10 min instead of the 60% PTFE solution.

Preparation of PTFE-dip coated CFP

Carbon-based electrodes were prepared by first ultrasonically cleaning 2.5 x 2.5 cm^2 CFP (Sigracet 29AA, Fuel Cell store) substrates in de-ionized water using an Emmi-30 HC sonicator. The CFP was pre-treated by UV/ozone (60 °C, 45 min). The 190 μm thick as-treated CFP was soaked horizontally into 60 wt% PTFE aqueous solution for 10 min and dried vertically at 120 °C under ambient conditions for 1 hour. Next, the dip coated CFP samples were annealed in a tube oven at 350 °C under argon atmosphere for 30 min.

Electrochemistry

Electrochemical experiments on pristine and PTFE coated nickel foam and carbon fibre paper electrocatalysts were run at room temperature in a customized gas-tight H-type PMMA cell (Area = 3.14 cm^2). Cell components were cleaned with 0.1 M H_2SO_4 and left in lightly boiling H_2O for 20 min, after it was rinsed thoroughly with ultrapure water. Cathodic and anodic compartments were separated by a Nafion 115 (FuelCellStore) cation-exchange membrane to prevent H_2O_2 degradation by cathodic reduction. A Solartron Analytical potentiostat was employed to control and record the electrochemical response. In a three-electrode configuration, the anodic compartment contained the nickel or carbon working electrode and a silver/silver chloride reference electrode (XR300, Radiometer Analytical), while a Pt mesh was used as the counter electrode. An aqueous solution of 1.0 M K_2CO_3 (pH 12.97) was used as an electrolyte, and the anolyte was stirred at a rate of 500 rpm during electrolysis. Formed H_2O_2 was stabilized by addition of 4 mg/ml Na_2SiO_3 (Sigma-Aldrich) to the electrolyte. The working electrode was subjected to a range of different potentials and were converted to the RHE scale using $E_{RHE} = E_{Ag/AgCl} + E°_{Ag/AgCl} + 0.059 \times pH$, where $E°_{Ag/AgCl}$ is the standard potential of Ag/AgCl at 25°C (0.197 V) and pH values were determined by a calibrated pH meter.

For measuring H_2O_2 concentrations a 0.02 M $KMnO_4$ (standardized against sodium thiosulfate, Titripur, reag. Ph. Eur., Sigma Aldrich) solution is used as the titrant. Concentrated sulphuric acid (95.7 wt%, Fisher Scientific) is diluted by addition to Milli-Q until a volumetric ratio of 1:1 is achieved. The solution was then cooled down to room temperature by immersing the flask in a bath of cold water. A 3 mL aliquot of electrolyte was added to a 50 mL beaker. Next, 15 mL of dilute H_2SO_4 was slowly added to the aliquot. The $KMnO_4$ titrant was added dropwise to the stirred solution using a burette. After a permanent light pink was apparent in the stirred solution the added volume of titrant was calculated and the procedure was repeated thrice for accuracy determination. Given the measured volume of titrant V_{EP} to reach the endpoint, the accumulated H_2O_2 concentration C_{H2O2} (ppm) follows from the reaction stoichiometry between H_2O_2 and MnO_4^-, as shown below :

$$2KMnO_4 + 5H_2O_2 + 3\,H_2SO_4 \rightarrow K_2SO_4 + 2\,MnSO_4 + 8\,H_2O + 5\,O_2$$

$$C_{H_2O_2} = \frac{(5/2) \cdot 34.015 \cdot V_{EP} \cdot C_{KMnO_4}}{V_{Al}}$$

where C_{KMnO4} is the concentration of titrant (mol L-1), V_{Al} is the volume of the electrolyte aliquot (L), and 34.015 is the molar mass of H_2O_2 (g mol-1).

X-Ray Photoelectron Spectroscopy

The XPS spectra were generated by Al and Mg Kα X-ray sources. A Thermo Scientific K-alpha apparatus equipped a Flood Gun for charge compensation of the sample was used as an Al K-alpha X-ray source. Parameters used for the measurements were: spot size of 400 µm, pass energy of 50 eV, energy step size of 0.1 eV and a dwell time of 50 ms. X-ray photoelectron analysis was carried out using a PHI-TFA XPS spectrometer (Physical Electronic Inc.) for Mg-monochromatic measurements. The vacuum during XPS analysis was 10^{-9} mbar. The analyzed area was 0.4 mm in diameter and the analysis depth was ~ 3 5 nm. Narrow multiplex scans of the peaks were recorded using a pass energy of 35 eV with a step size 0.5 eV, at a take-off angles of 45° with respect to the sample surface. Low energy electron gun was used for surface charge neutralization XPS. Spectra were processed using Multipak v8.0 (Physical Electronics Inc.).

Density Functional Theory calculations

β-NiOOH DFT+U Calculations.

All spin-polarized density functional theory (DFT) calculations in the present work have been carried out within the periodic plane wave framework as implemented in Vienna Ab initio Simulation Package (VASP) version 5.4.1. The DFT+U correction method of Dudarev et al.[1] was employed to ameliorate the known deficiencies when describing partially occupied 3d shells with the traditional approach. A U-J value of 5.5 eV for Ni(III) was added in combination with the Perdew-Burke-Ernzerhof (PBE) exchange-correlation functional. This value was adapted from the linear response theory calculations of Li and Selloni[2] on β-NiOOH and has been confirmed to lead to accurate replication of electronic and structural properties among other parameter values by Carter et al.[3] Blöchl's all-electron, frozen-core, projector augmented wave (PAW) method was used. The initial β-NiOOH structure (that was subsequently optimized) was taken from the literature.[4] Convergence to within less than 1 meV/atom for the total energies is achieved using a plane-wave kinetic energy cut-off of 500 eV and a Γ-point-centered Monkhorst-Pack k-point mesh of size 3 x 3 x 1 for unit cell calculations. Geometry optimizations were carried out until the forces on all atoms were less than 0.01 eV/Å. The positions of all atoms were allowed to relax but lattice parameters were fixed to their equilibrium bulk values. Gaussian smearing with a width of 0.01 eV was used to aid the convergence of the calculations. Non-converging situations were helped by switching from Pulay mixing with an initial approximation for the charge dielectric function according to Kerker, to a linear mixing algorithm. Brillouin zone integration using the tetrahedron method with Blöchl corrections was used subsequently to evaluate the final energies. Moreover, dipole corrections were applied in the direction perpendicular to the slab. For all of the analyses, assessment of the different models and graphical representation of the structures obtained were carried out with Visualization for Electronic and Structural Analysis (VESTA) software codes.

The β-NiOOH and β-NiOOH-CF3 unit cells

Unit cell calculations were performed on a stoichiometric slab model with half of the surface oxygen atoms hydroxylated ($Ni_{16}O_{31}H_{16}$). Rossmeisl et al. found that the OER overpotential on

RuO2 was lower on an O-terminated slab than on an OH-terminated slab.[5] Because NiOOH is not stable for a fully O-terminated slab, we instead assessed the OER performance on a surface with less than full hydroxylation. The active site on the oxide catalyst that allowed formation of the different adsorbed intermediates was a coordinatively unsaturated site on one side of the slab in the unit cell. To chemically bind the short-polymer CF_3 to the metal surface a terminating OH group was removed from a Ni site on the same side as the unsaturated Ni. Slabs of four layers were used for all surface calculations. The resulting relaxed unit cells for β-NiOOH is displayed in Figure S5.

Computational water oxidation mechanisms

At variance with the numerous experimental investigations, computational studies on the two-electron H_2O-to-H_2O_2 reaction are still scarce. Most studies in this domain consider the OH* intermediate as the sole intermediate and descriptor for catalyst activity: weak OH* free energy will lead to a high preference towards the two-electron route.[6,7] The associative mechanism for the four-electron water oxidation involves five elementary steps: one thermochemical step (eq. 1) and four electrochemical steps (eq. 2-5), where the * symbol used refers to a unique free catalytic site. After thermochemical water adsorption at the active site to form H_2O*, the intermediate undergoes deprotonation and proton coupled electron transfer (PCET) to form OH* species with production of a H^+/e^- pair. Formed OH* is converted to O* by a following PCET, and addition of a water molecule and a PCET event converts the O* species to OOH*. Finally, O_2 gas and the fourth H^+/e^- is produced from a last PCET step. Protons ejected into the electrolyte will eventually meet a transferring electron at the cathode. The desorption of O_2 – which is a thermochemical step – has been omitted because GGA tends to fail miserably at describing gas-phase reaction barriers and describing the energetics of O_2* along its reaction coordinate.[8,9]

$$* \; + \; H_2O\,(l) \rightarrow H_2O^* \qquad (1)$$

$$H_2O^* \rightarrow OH^* \; + \; (H^+ \; + \; e^-) \qquad (2)$$

$$OH^* \rightarrow O^* + (H^+ \; + \; e^-) \qquad (3)$$

$$O^* \; + \; H_2O\,(l) \rightarrow OOH^* \; + \; (H^+ \; + \; e^-) \qquad (4)$$

$$OOH^* \rightarrow O_2 \; + \; (H^+ \; + \; e^-) + \; * \qquad (5)$$

Considering the associative mechanism, the equation for the two-electron H_2O_2 production consistently used involves the addition of a water molecule and a PCET event that converts the OH intermediate (eq. 6).[10,11] Next to this so-called Volmer-Heyrovksi mechanism, another possibility was considered. Repetition of the Volmer step and direct coupling of the two adsorbed OH* intermediates at the surface is referred to as the Volmer-Tafel mechanism (eqn. 7). These two mechanisms thus differ in the sequence by which the second water molecule is deprotonated. Due to the size induced limitation of our slabs, only Volmer-Heyrovski mechanism is considered in this work.

$$OH^{*} + H_2O\,(l) \rightarrow H_2O_2\,(l) + (H^{+} + e^{-}) + * \qquad \text{(Volmer-Heyrovski)} \qquad (6)$$

$$OH^{*} + OH^{*} \rightarrow H_2O_2\,(l) \qquad \text{(Volmer-Tafel)} \qquad (7)$$

The trends for water oxidation chemistry can, thus, be described by the binding of four key intermediates: H_2O^*, OH^*, O^*, and OOH^*. Clearly, after adsorption of the OH intermediate the water oxidation could either evolve towards complete four-electron oxygen evolution or two-electron peroxide generation. It should be noted here that all considered electrochemical steps have been written in the "acidic" convention where each equation consists of the production of a proton rather than the consumption of a hydroxide ion. For the present thermodynamic study, the acidity or basicity of the environment has no ramifications for the equilibrium constants of each of the electrochemical steps as they only depend on the potential versus RHE (U_{RHE}).

Thermodynamic intermediate free energy calculations

From a modelling point of view, DFT allows simple calculation of the free energies of surface adsorbed species but determining the chemical potentials of the removed protons and electrons imagined as being put into a reservoir is much less straightforward. As a remedy to this dilemma, Rossmeisl and Nørskov put forward the idea of following the experimental comparison route used for electrochemical performances: aligning potentials with respect to a reference reaction.[5] Catalytic activity for OER on the NiOOH active sites was characterized by calculating the Gibbs free energies of the individual reaction steps using the standard hydrogen electrode (SHE) as a reference electrode. The acidic, associative mechanism has four elementary steps that involve electron and proton transfer processes ($H^+ + e^-$), and their Gibbs free energy is usually calculated implicitly by assuming the equilibrium $H^+ + e^- \leftrightarrow \frac{1}{2} H_2$.[12] The effect of the electrode potential U_{SHE} on all electrochemical steps releasing an electron in the electrode is included by shifting the Gibbs free energy by $\Delta G_U = -eU$. At standard conditions (pH = 0, pressure pH_2 = 1 bar, and T = 298 K) the reaction free energy is zero at an applied potential of U = 0. Then, the free energy $\Delta G_0 = \Delta G(U = 0, pH = 0, p = 1\text{ bar}, T)$ of the reaction $*AH \rightarrow A + H^+ + e^-$, can be calculated as the free energy of the reaction $AH \rightarrow A + \frac{1}{2} H_2$. The free energy of $H^+ + e^-$ in solution, thus, equals that of $\frac{1}{2} H_2$ in the gas phase.

The reaction Gibbs free energies, ΔG_n corresponding to equations (1)-(5), which provide the binding strength between the NiOOH catalyst and OER intermediates, are shown in equations (8)-(12). Calculated values for the reaction free energy ΔE are corrected by including zero-point energies due to reaction (ΔZPE), the change in entropy (TΔS), and enthalpy correction (ΔH) (i.e., $\Delta G_n = \Delta E + \Delta ZPE - T\Delta S - \Delta H$). The total Gibbs free energy of the four-electron water oxidation reaction $2H_2O \rightarrow O_2 + 2H_2$ is fixed at the experimental value (4.92 eV) to avoid the need for performing energy calculations on O_2, since using DFT with a GGA functional is erroneous in accurately describing the electronic structure and energy of the molecule.[5]

$$\Delta G_1 = E_{H_2O*} - E_{*} - E_{H_2O} + (\Delta ZPE - T\Delta S + \Delta H)_1 \qquad (8)$$

$$\Delta G_2 = E_{OH*} + \frac{1}{2} E_{H_2} - E_{H_2O*} + (\Delta ZPE - T\Delta S + \Delta H)_2 - eU \qquad (9)$$

$$\Delta G_3 = E_{O*} + \frac{1}{2}E_{H_2} - E_{OH*} + (\Delta ZPE - T\Delta S + \Delta H)_3 - eU \quad (10)$$

$$\Delta G_4 = E_{OOH*} + \frac{1}{2}E_{H_2} - E_{O*} - E_{H_2O} + (\Delta ZPE - T\Delta S + \Delta H)_4 - eU \quad (11)$$

$$\Delta G_5 = 4.92 - \Delta G_1 - \Delta G_2 - \Delta G_3 - \Delta G_4 + (\Delta ZPE - T\Delta S + \Delta H)_5 \quad (12)$$

where $E_*, E_{H_2O*}, E_{OH*}, E_{O*}, E_{OOH*}$ are the total energies of the clean surface (*) and of surfaces with the single adsorbed species OH, O, and OOH, respectively, and E_{H_2O} and E_{H_2} are the total energies of H_2O and H_2 molecules, all obtained from DFT calculations. $(\Delta ZPE - T\Delta S)_n$ (n = 1, 2, 3, 4) can be calculated as:

$$(\Delta ZPE - T\Delta S + H)_1 = (ZPE_{H_2O*} - TS_{H_2O*} + H_{H_2O*}) - (ZPE_* - TS_* + H_*) - (ZPE_{H_2O} - TS_{H_2O} + H_{H_2O}) \quad (13)$$

$$(\Delta ZPE - T\Delta S + H)_2 = (ZPE_{OH*} - TS_{OH*} + H_{OH*}) - (ZPE_{H_2O} - TS_{H_2O} + H_{H_2O}) + \frac{1}{2}(ZPE_{H_2} - TS_{H_2} + H_{H_2}) \quad (14)$$

$$(\Delta ZPE - T\Delta S + H)_3 = (ZPE_{O*} - TS_{O*} + H_{O*}) - (ZPE_{OH*} - TS_{OH*} + H_{OH*}) + \frac{1}{2}(ZPE_{H_2} - TS_{H_2} + H_{H_2}) \quad (15)$$

$$(\Delta ZPE - T\Delta S + H)_4 = (ZPE_{OOH*} - TS_{OOH*} + H_{OOH*}) - (ZPE_{O*} - TS_{O*} + H_{O*}) + \frac{1}{2}(ZPE_{H_2} - TS_{H_2} + H_{H_2}) \quad (16)$$

Zero-point energies and thermal corrections were calculated using the harmonic oscillator approximation at standard state conditions (298 K) to evaluate vibrational enthalpic and entropic terms for the adsorbates once bound to the surface. ZPEs and thermal contributions for the adsorbates prior to adsorption were taken from the literature and originate from ideal gas, rigid rotor, and harmonic oscillator approximations to evaluate respectively translational, rotational, and vibrational terms. Numerical Hessian matrices were constructed from finite differences of displacement and force components on each atom. The adsorbed intermediate, as well as the Ni/O/H atoms and polymer fragment in first layer nearest to the reaction intermediate, were displaced by ±0.02 Å in all three directions from their equilibrium positions. The resulting Hessian matrix then was diagonalized to yield the vibrational frequencies v_j corresponding to each normal mode j. By applying statistical thermodynamics based on the harmonic oscillator model the vibrational contributes are given by equations (18)-(19). Enthalpy changes due to temperature increase from 0 to 298 K are expected to be small and are normally neglected in calculations (assuming ΔH(298 K) = ΔH(0 K))[13,14], but are considered here because the energy reported by Carter et al. for the pristine slab has been used in this model.

$$ZPE = \frac{1}{2}\sum_j v_j \quad (17)$$

$$S_{vib} = \sum_j \left[\frac{v_j}{T\left(\exp\left(\frac{v_j}{kT}\right) - 1\right)} - k\ln\left(1 - \exp\left(-\frac{v_j}{kT}\right)\right) \right] \quad (18)$$

$$[H(T) - H(0)]_{vib} = \sum_j \frac{v_j}{\exp\left(\frac{v_j}{kT}\right) - 1} \quad (19)$$

where k is the Boltzmann constant and T is the temperature. Zero-point vibrational energy correction is required to obtain the true zero-temperature energy of the system.

Following scaling relations were used in this work :

$$\Delta G_{O^*} = 2\Delta G_{OH^*} + 0.28\ (eV) \quad (20)$$

$$\Delta G_{OOH^*} = \Delta G_{OH^*} + 3.2\ (eV) \quad (21)$$

Lastly, following a similar approach for obtaining free energies and energy corrections, the effective adsorption energies of key OER intermediate species at the surface O vacancy site, ΔE_i (i = OH*, O*, and OOH*), were calculated relative to H_2O and H_2 (at U = 0 and pH = 0)[13,15] :

$$\Delta E_{OH*} = E_{OH*} - E_* - E_{H_2O} + \frac{1}{2}E_{H_2} + (\Delta ZPE - T\Delta S + \Delta H)_A \quad (22)$$

$$\Delta E_{O*} = E_{O*} - E_* - E_{H_2O} + E_{H_2} + (\Delta ZPE - T\Delta S + \Delta H)_B \quad (23)$$

$$\Delta E_{OOH*} = E_{OOH*} - E_* - 2\,E_{H_2O} + \frac{3}{2}E_{H_2} + (\Delta ZPE - T\Delta S + \Delta H)_B \quad (24)$$

Bader charge analysis

Electron density calculations were performed within the density functional theory framework. The calculated electron charge density from the relaxed atomic structure was used to perform Bader charge analysis to investigate the electron transfer nature and electronic interactions for Ni-CF_3 adsorbate systems, using the code developed by Henkelman and co-workers.[16] This algorithm is based on the Bader partitioning scheme where properties of the condensed phase system are decomposed into contributions from the individual atoms.

Electrochemical measurements

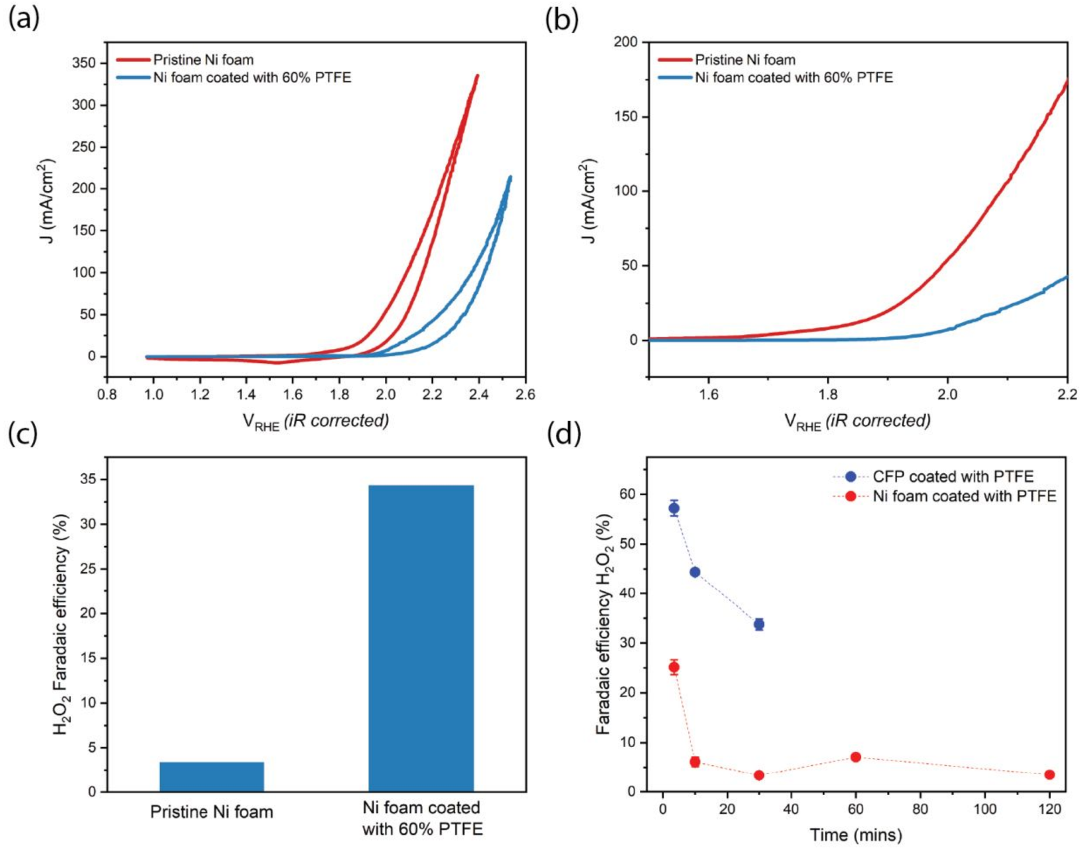

Figure S1 : (a) Cyclic voltammetries of the pristine and PTFE coated nickel foam in 1M K_2CO_3. (b) Zoomed in image of this cyclic voltammetry showing the delayed onset potential of water oxidation in PTFE coated nickel foam electrode. (c) Plot showing the improved faradaic efficiency towards H_2O_2 on the PTFE coated nickel foam electrode. (d) Degradation of the H_2O_2 faradaic efficiency over time on PTFE coated nickel and CFP electrodes.

The pristine and PTFE coated Ni foam were tested in an H-cell with 1 M K_2CO_3 as the electrolyte. The polarisation curves for each are shown in Figure S1 (a). Two key differences can be observed here. First, the performance of the PTFE coated Ni foam electrode is inferior to the pristine Ni foam electrode. This is a result of the passivation of the active sites in the Ni foam by the insulating

PTFE. As a result, the electrochemically active surface area is less on the PTFE coated Ni foam in comparison to the pristine Ni foam electrodes. This was also observed by Xia et al.[17] with their PTFE coated Carbon Fiber Paper (CFP) electrodes. The second key difference is in the delay of the onset potential for the PTFE coated Ni foam electrode. This is clear when looking at the magnified image of the polarisation curves, as shown in Figure S1 (b). The delayed onset potential of the PTFE coated electrode is an indication of a higher overpotential for the thermodynamically favourable water oxidation reaction, i.e., the four electron water oxidation to oxygen. This higher overpotential suggests that the PTFE modification has made the Ni foam less active towards the oxygen evolution reaction. The redox wave pertaining to the potential induced transition of Ni^{2+} to Ni^{3+} is also visible in the voltammogram in Figure S1 (a).

Next the electrodes were tested for its selectivity towards the two electron water oxidation reaction to hydrogen peroxide. A chronoampherometry was performed until a fixed amount of charge was passed through the setup and the hydrogen peroxide produced at the anode was quantified using permanganate titration. The hydrogen peroxide faradaic efficiency of the pristine and the PTFE coated Ni foam electrode are shown in Figure S1 (c). As expected, the faradaic efficiency towards hydrogen peroxide by the PTFE coated Ni foam electrode was higher compared to the pristine Ni foam electrode, agreeing with the work of Xia et al.[17], suggesting that the PTFE modification has indeed altered the selectivity of the water oxidation reaction. We noticed a faster decrease of this faradaic efficiency over time compared to the work of Xia et al., as shown in Figure S1 (d). This is due to the higher surface area of the electrodes (3.14 cm^2) used in our work, compared to the work of Xia et al. (0.42 cm^2) as well as the lower amount of anolyte in our H cell compared to that of Xia et al. Higher surface area and higher concentrations of hydrogen peroxide will lead to the accelerated degradation of the hydrogen peroxide via its oxidation at the anode, as shown in equation 27. This oxidative decay of the hydrogen peroxide to oxygen with time, the oxygen bubble trapping nature of the porous structure of the Ni foam and the aerophilic character of the PTFE makes the oxygen gas quantification unreliable and hence is not presented here. To confirm that the decay observed here is from the hydrogen peroxide degradation and not the sample itself, we performed repeat experiments on the same sample by replacing the electrolyte. As shown in Figure S2, similar hydrogen peroxide faradaic efficiency was recovered by performing the electrolysis with a fresh electrolyte, confirming that it is indeed the hydrogen peroxide degradation that is resulting in drop in faradaic efficiency with time. For best results, it would be ideal to minimize the residence time of the hydrogen peroxide in the anodic chamber either by using a flow cell or through other approaches.

$$O_2 + 4H^+ + 4e^- \rightarrow 2H_2O \;, \quad E^o = 1.23\ V\ vs\ NHE \qquad (24)$$

$$H_2O_2 + 2H^+ + 2e^- \rightarrow 2H_2O \;, \quad E^o = 1.77\ V\ vs\ NHE \qquad (25)$$

$$OH^{\cdot} + H^+ + e^- \rightarrow H_2O \;, \quad E^o = 2.38\ V\ vs\ NHE \qquad (26)$$

$$O_2 + 2H^+ + 2e^- \rightarrow H_2O_2 \;, \quad E^o = 0.695\ V\ vs\ NHE \qquad (27)$$

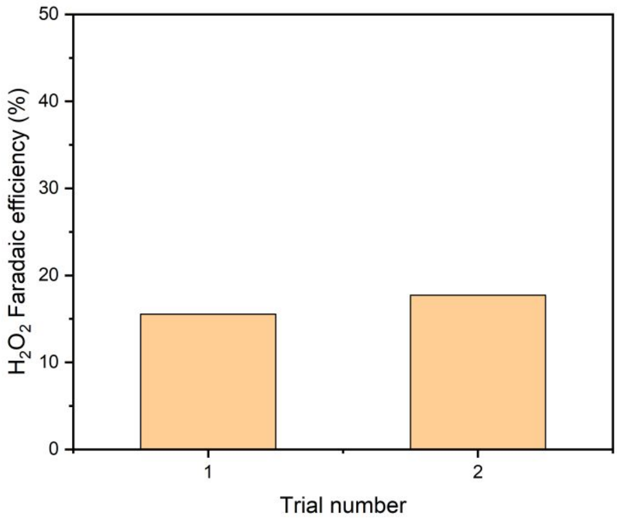

Figure S2 : Repeat chronoampherometric tests on the same sample, with fresh electrolyte, shows that similar faradic efficiencies can be regained. This again suggests that the drop in faradic efficiency seen earlier is through H_2O_2 degradation at the anode and not because of sample degradation.

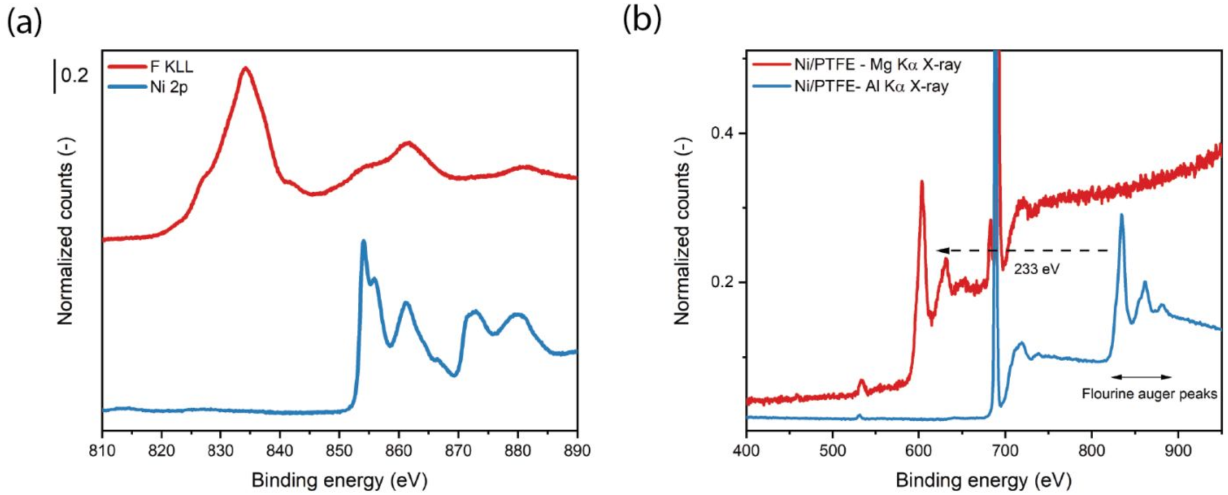

Figure S3 : (a) Ni2p core electron and F KLL auger emission spectra acquired using an XPS system with Al Kα X-ray source, showing their obvious overlap. (b) Survey spectra of the PTFE coated Ni foam samples using XPS systems with Al Kα (1486.6 eV) and Mg Kα (1253.6 eV) X-ray sources. As shown in the figure, the F KLL auger emission spectra is shifted to lower binding energies when using a Mg Kα X-ray source, by a factor equalling the difference between the X-ray energies.

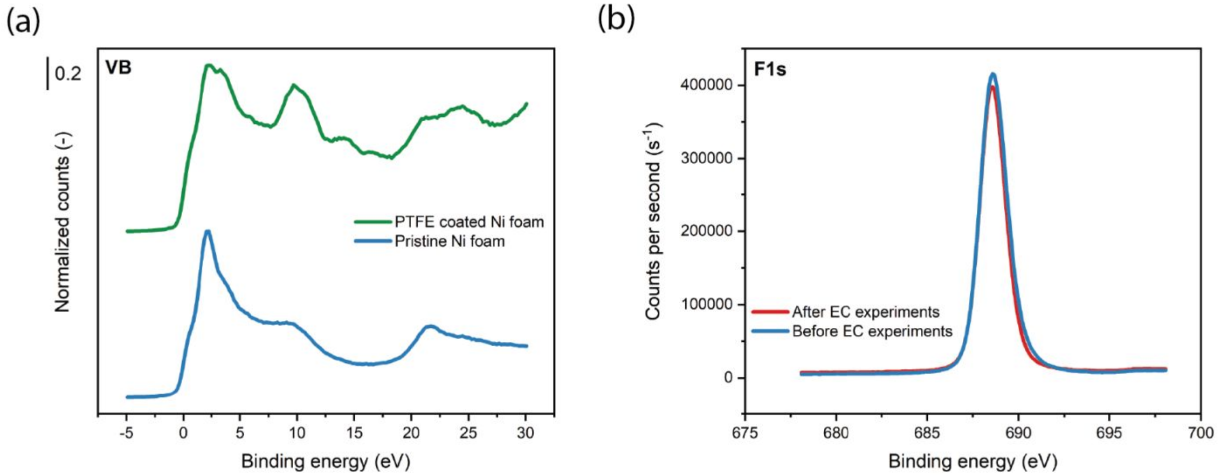

Figure S4 : (a) Valence band XPS spectra of the pristine and PTFE coated Ni foam electrodes. (b) F1s core electron spectra of the PTFE coated Ni foam electrode before and after electrochemical experiments, again showing the PTFE coating was stable during the electrochemical experiments.

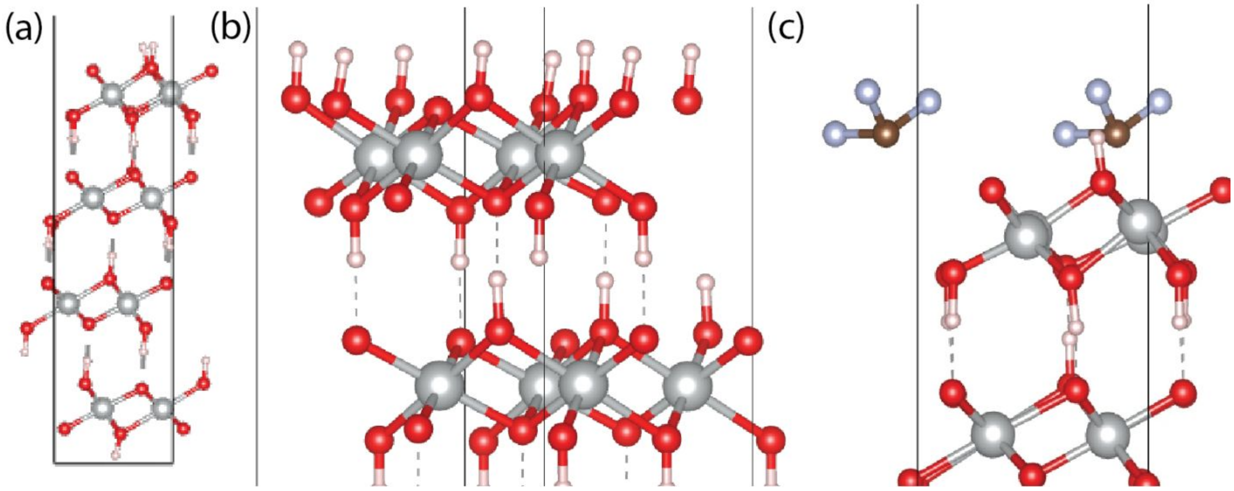

Figure S5 : (a-b) Relaxed β-NiOOH unit cells used in this work (c) Relaxed β-NiOOH-CF_3 unit cells used in this work

Table S1 : Values of the zero-point energies, enthalpic, and entropic contributions for the OER

		ZPE (eV)	H (eV)	TS (eV)	Total correction (eV)
β-NiOOH	H_2O*	2.18	0.04	0.05	2.17
	OH*	1.85	0.09	0.13	1.82
	O*	1.64	0.08	0.11	1.61
β-NiOOH-CF_3	H_2O*	2.21	0.07	0.09	2.19
	OH*	1.84	0.08	0.11	1.82
	O*	1.59	0.09	0.12	1.56

Table S2: Mechanism reaction energetics on the NiOOH surface. (Reaction free energies include the ZPE and thermal energy corrections.)

		Reaction energies (eV)	Reaction free energies (eV)
H_2O/H_2O^* without CF_3	H_2O + * → H_2O*	-0.86	-0.35
H_2O/H_2O^* with CF_3		-0.64	0.20
H_2O^*/OH^* without CF_3	H_2O* → OH* + (H^+ + e^-)	0.85	0.43
H_2O^*/OH^* with CF_3		0.47	0.02
OH^*/O^* without CF_3	OH* → O* + (H^+ + e^-)	1.60	1.32
OH^*/O^* with CF_3		1.70	1.37
O^*/OOH^* without CF_3	O* + H_2O → OOH* + (H^+ + e^-)	1.60	1.88
O^*/OOH^* with CF_3		1.50	1.82
OOH^*/O_2 without CF_3	OOH* → O_2 + * + (H^+ + e^-)	1.72	1.63
OOH^*/O_2 with CF_3		1.88	1.50

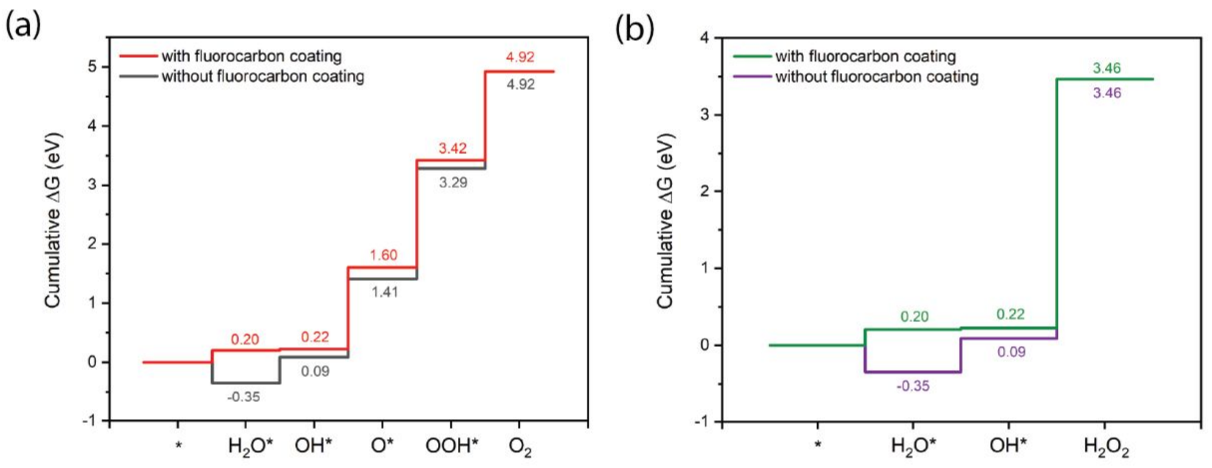

Figure S6 : (a) Cumulative free energy profile for the four electron water oxidation reaction to oxygen for β-NiOOH and β-NiOOH-CF_3 unit cells (associate pathway) (b) Cumulative free energy profile for the two electron water oxidation reaction to hydrogen peroxide for β-NiOOH and β-NiOOH-CF_3 unit cells (Volmer Heyrovski pathway)

Table S3 : Bader net atomic charges for β-NiOOH and β-NiOOH-CF_3 and OH^* intermediate

	**NiOOH-OH<br>Bader net atomic charge**
Ni	1.241
Ni	1.199
Ni	1.084
Ni	1.206
Ni	1.231
Ni	1.183
Ni	1.079
Ni	1.142
Ni	1.149
Ni	1.882
Ni	1.121
Ni	1.254
Ni	1.135
Ni	1.403
Ni	0.851
Ni	1.252
O	-0.659
O	-0.644
O	-0.743
O	-0.72
O	-0.639
O	-0.641
O	-0.747
O	-0.716

	**NiOOH-OH-CF3<br>Bader net atomic charge**
Ni	0.85
Ni	1.338
Ni	1.349
Ni	1.015
Ni	0.838
Ni	1.331
Ni	1.362
Ni	1.132
Ni	1.279
Ni	1.401
Ni	1.101
Ni	1.231
Ni	1.294
Ni	1.428
Ni	1.137
Ni	1.235
O	-0.784
O	-0.631
O	-0.633
O	-0.732
O	-0.811
O	-0.676
O	-0.627
O	-0.748

O	-0.569	O	-0.755
O	-0.823	O	-0.615
O	-0.751	O	-0.685
O	-0.749	O	-0.584
O	-0.739	O	-0.684
O	-0.742	O	-0.574
O	-0.779	O	-0.746
O	-0.622	O	-0.616
O	-0.711	O	-0.503
O	-1.006	O	-0.697
O	-0.625	O	-0.698
O	-0.741	O	-0.63
O	-0.651	O	-0.508
O	-0.986	O	-0.712
O	-0.648	O	-0.68
O	-0.494	O	-0.678
O	-0.746	O	-0.685
O	-0.667	O	-0.753
O	-0.696	O	-0.777
O	-0.659	O	-0.674
O	-0.755	O	-0.693
O	-0.659	O	-0.74
O	-0.758	O	-0.769
O	-5.708	H	0.06
H	0.164	H	0.23
H	0.501	H	0.155
H	0.097	H	0.103
H	0.038	H	0.126
H	0.079	H	0.229
H	0.502	H	0.165
H	0.09	H	0.074
H	0.098	H	0.189
H	0.183	H	0.178
H	0.328	H	0.15
H	0.264	H	-0.042
H	0.191	H	0.157
H	0.254	H	-0.076
H	0.168	H	0.166
H	0.174	H	0.208
H	0.118	C	1.235
H	0.131	F	-0.511
		F	-0.402
		F	-0.618

References

1. Dudarev, S. & Botton, G. Electron-energy-loss spectra and the structural stability of nickel oxide: An LSDA+U study. *Phys. Rev. B - Condens. Matter Mater. Phys.* **57**, 1505–1509

(1998).

2. Li, Y. F. & Selloni, A. Mechanism and activity of water oxidation on selected surfaces of pure and Fe-Doped NiOx. *ACS Catal.* **4**, 1148–1153 (2014).

3. Tkalych, A. J., Yu, K. & Carter, E. A. Structural and Electronic Features of β-Ni(OH)2 and β-NiOOH from First Principles. *J. Phys. Chem. C* **119**, 24315–24322 (2015).

4. Tkalych, A. J., Zhuang, H. L. & Carter, E. A. A Density Functional + U Assessment of Oxygen Evolution Reaction Mechanisms on β-NiOOH. *ACS Catal.* **7**, 5329–5339 (2017).

5. Rossmeisl, J., Logadottir, A. & Nørskov, J. K. Electrolysis of water on (oxidized) metal surfaces. *Chem. Phys.* **319**, 178–184 (2005).

6. Siahrostami, S., Li, G. L., Viswanathan, V. & Nørskov, J. K. One- or Two-Electron Water Oxidation, Hydroxyl Radical, or H2O2 Evolution. *J. Phys. Chem. Lett.* **8**, 1157–1160 (2017).

7. Shi, X. *et al.* Understanding activity trends in electrochemical water oxidation to form hydrogen peroxide. *Nat. Commun.* **8**, 6–11 (2017).

8. Dickens, C. F., Kirk, C. & Nørskov, J. K. Insights into the electrochemical oxygen evolution reaction with ab initio calculations and microkinetic modeling: Beyond the limiting potential volcano. *J. Phys. Chem. C* **123**, 18960–18977 (2019).

9. Gerrits, N. *et al.* Density Functional Theory for Molecule–Metal Surface Reactions: When Does the Generalized Gradient Approximation Get It Right, and What to Do If It Does Not. *J. Phys. Chem. Lett.* **11**, 10552–10560 (2020).

10. Viswanathan, V., Hansen, H. A. & Nørskov, J. K. Selective Electrochemical Generation of Hydrogen Peroxide from Water Oxidation. *J. Phys. Chem. Lett.* **6**, 4224–4228 (2015).

11. Nadar, A. *et al.* Evaluating the Reactivity of BiVO4 Surfaces for Efficient Electrocatalytic H2O2 Production: A Combined Experimental and Computational Study. *J. Phys. Chem. C* **124**, 4152–4161 (2020).

12. Sinha, V., Sun, D., Meijer, E. J., Vlugt, T. J. H. & Bieberle-Hutter, A. A multiscale modelling approach to elucidate the mechanism of the oxygen evolution reaction at the hematite-water interface. *Faraday Discuss.* **229**, 89–107 (2021).

13. Liao, P., Keith, J. A. & Carter, E. A. Water oxidation on pure and doped hematite (0001) surfaces: Prediction of Co and Ni as effective dopants for electrocatalysis. *J. Am. Chem. Soc.* **134**, 13296–13309 (2012).

14. Valdes, A., Qu, Z. W., Kroes, G. J., Rossmeisl, J. & Nørskov, J. K. Oxidation and Photo-Oxidation of Water on TiO2 Surface. *J. Phys. Chem. C.* **112**, 9872–9879 (2008).

15. Man, I. C. *et al.* Universality in Oxygen Evolution Electrocatalysis on Oxide Surfaces. *ChemCatChem* **3**, 1159–1165 (2011).

16. Tang, W., Sanville, E. & Henkelman, G. A grid-based Bader analysis algorithm without lattice bias. *J. Phys. Condens. Matter* **21**, 084204 (2009).

17. Xia, C. *et al.* Confined local oxygen gas promotes electrochemical water oxidation to hydrogen peroxide. *Nat. Catal.* **3**, 125–134 (2020).
